# Cortical Sensitivity to Visual Features in Natural Scenes

**DOI:** 10.1371/journal.pbio.0030342

**Published:** 2005-09-27

**Authors:** Gidon Felsen, Jon Touryan, Feng Han, Yang Dan

**Affiliations:** **1** Division of Neurobiology, Department of Molecular and Cell Biology, University of California, Berkeley, California, United States of America,; **2** Helen Wills Neuroscience Institute, University of California, Berkeley, California, United States of America,; **3** Group in Vision Science, University of California, Berkeley, California, United States of America; Istituto di NeurofisiologiaItaly

## Abstract

A central hypothesis concerning sensory processing is that the neuronal circuits are specifically adapted to represent natural stimuli efficiently. Here we show a novel effect in cortical coding of natural images. Using spike-triggered average or spike-triggered covariance analyses, we first identified the visual features selectively represented by each cortical neuron from its responses to natural images. We then measured the neuronal sensitivity to these features when they were present in either natural images or random stimuli. We found that in the responses of complex cells, but not of simple cells, the sensitivity was markedly higher for natural images than for random stimuli. Such elevated sensitivity leads to increased detectability of the visual features and thus an improved cortical representation of natural scenes. Interestingly, this effect is due not to the spatial power spectra of natural images, but to their phase regularities. These results point to a distinct visual-coding strategy that is mediated by contextual modulation of cortical responses tuned to the spatial-phase structure of natural scenes.

## Abstract 2

By recording how visual cortical neurons respond to natural scenes versus random stimuli, the authors discover that complex cells, but not simple cells, respond more sensitively to the phase regularities of natural images

## Introduction

An essential goal in studying the visual system is to understand how it processes natural scenes, which exhibit distinct statistical properties [1–7]. In particular, since neuronal circuits evolve and develop in the natural environment, they may be specifically adapted for efficient coding of natural stimuli [1–4,8–10]. This efficient coding hypothesis has provided an important framework for understanding early visual processing: In the retina and the lateral geniculate nucleus, spatiotemporal frequency tuning of the neurons appears to be adapted to the power spectra of natural scenes, allowing them to encode the input into a more efficient, decorrelated form [3,11–13]. In the primary visual cortex (V1), response properties of both simple [14–16] and complex [17,18] cells can be “derived” from the statistics of natural scenes and the principle of efficient coding. These studies underscore the importance of understanding neuronal response properties with respect to natural-scene statistics.

The function of neurons in the early visual pathway is thought to be the analysis of local features in the images. The response of each neuron is determined by two properties: the structure of its preferred visual features and the sensitivity of the neuron to the presence of these features in visual scenes. The preferred features are closely related to the receptive field (RF) of the cell. For a neuron with linear RF properties (e.g., a simple cell), the preferred feature directly corresponds to the classical RF (i.e., light and dark regions correspond to ON and OFF response regions). In a standard model of the complex-cell RF, there are multiple preferred features, and each feature corresponds to the RF of a functional subunit [19–21] or a linear combination of them. Feature sensitivity, on the other hand, can be characterized by the contrast-response function, which describes the relationship between the neuronal response and the contrast of the feature (see below). High feature sensitivity allows the neuron to reliably signal the presence of the preferred features in visual stimuli. Note that, in this study, we use the term “preferred feature” solely to facilitate the discussion of our findings on cortical feature sensitivity; we do not imply that these features are necessarily optimal for driving the cortical neuron, as the neuronal responses are known to be modulated by various contextual stimuli, including those in the nonclassical RF [22].

The structure of the preferred features of V1 neurons has been investigated extensively in experimental studies [19,20,23,24], and is thought to play an important role in efficient, sparse coding of natural scenes [14–18]. However, the role of feature sensitivity in efficient coding has not been studied experimentally. In the present study, we measured feature sensitivity of cortical neurons in processing several classes of visual stimuli. By comparing the cortical feature sensitivity for natural images, random stimuli, and synthetic stimuli with either natural power or natural phase spectra, we characterized the dependence of feature sensitivity on the statistical properties of visual stimuli. Our results point to a novel form of efficient coding that is mediated by contextual modulation of cortical responses.

## Results

### Measurement of Preferred Features and Feature Sensitivity

**Figure 1:**
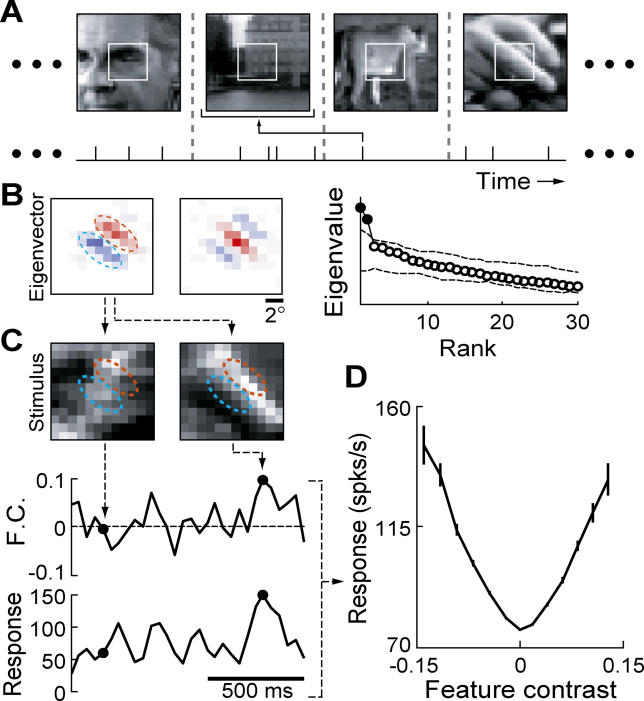
Measurement of Preferred Features and Feature Sensitivity for V1 Complex Cells (A) Upper panel: example natural images. White boxes (12 × 12 pixels) indicate area presented in experiments. Lower panel: schematic spike train, binned at stimulus frame rate (24 Hz, dotted lines). Arrow indicates temporal delay (1 frame) at which preferred features were estimated, which was determined in preliminary studies to be the optimal temporal delay (see Figure S2). (B) Estimation of preferred features (significant eigenvectors) using STC analysis (see Materials and Methods). Left panel: preferred features of a neuron, with light and dark regions represented by red and blue; dashed ovals delineate the first feature to facilitate comparison with the images. Right panel: 30 largest eigenvalues of STC matrix. Dashed lines: control confidence intervals (mean ± 12 standard deviation of control eigenvalues). Filled circles: significant eigenvalues corresponding to eigenvectors shown on left. (C) Upper panel: natural images. Dashed ovals correspond to those in (B). Middle panel: contrast of the first preferred feature (F.C. denotes feature contrast; see Materials and Methods). Lower panel: responses of the neuron (in spikes/s) to natural images. Black dots: feature contrasts (middle) and neuronal responses (lower) for the example images. (D) Contrast-response function. Error bar: ± standard error of the mean.

Single-unit recordings were made from 50 neurons (36 complex cells, 14 simple cells) in area 17 of anesthetized adult cat (see Materials and Methods). We will first report the findings from complex cells and will then describe the results from simple cells in a separate section. In order to characterize the feature sensitivity of each complex cell, it is necessary to first estimate its preferred features. The preferred features were estimated from the responses of the neuron to a natural-image ensemble (Figure 1A), using a spike-triggered covariance (STC) analysis [25–27] (see Materials and Methods). These features, which are represented by the “significant eigenvectors” of the STC matrix, contained oriented light and dark regions (Figure 1B), resembling the RFs of simple cells [19]. For the majority of the cells analyzed with this method (17/26), we identified two significant eigenvectors, which are well approximated by a pair of Gabor functions with similar orientations and spatial frequencies but ∼90° phase difference (Figure 1B, left panel). For other cells, only one significant eigenvector was identified, which was also well approximated by a Gabor function.

The contrast of each significant eigenvector in a particular image (referred to as “feature contrast”) was measured as the dot product of the image and the eigenvector (Figure 1C), which depends on both the overall contrast of the image and its similarity with the eigenvector (e.g., the image on the right in Figure 1C contains a high-contrast luminance edge matched to the first eigenvector, thus giving rise to a high feature contrast). The sensitivity of the neuron to each of its significant eigenvectors in a stimulus ensemble is measured by plotting the average neuronal response as a function of feature contrast (Figure 1D), yielding the contrast-response function [26,28,29] (see Materials and Methods). A steep contrast-response function indicates a high sensitivity of the neuron to the presence of the corresponding feature in the images, whereas a flat contrast-response function indicates that the neuronal response is insensitive to the presence of the feature.

The structure of the significant eigenvectors (Figure 1B) and the shape of their contrast-response functions (Figure 1D) are qualitatively consistent with the standard model of complex-cell RFs [19–21]. This “energy model” can be described as



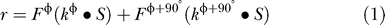



where *r* is the response of the neuron, *S* is the stimulus, *k* ^φ^ is the RF of a subunit with preferred spatial phase φ (*k* ^φ^, *k* ^φ+90°^, and their linear combinations also correspond to the preferred features of the cell), and *F* represents the contrast-response function for the subunit, which is commonly approximated by *F*(*x*) = β*x* ^2^. Given the quadratic nonlinearity in this model [21], the STC analysis provides an ideal method for estimating the preferred features of each complex cell: The RF of each subunit (*k* ^φ^ or *k* ^φ+90°^) corresponds to a significant eigenvector (Figure 1B) or a linear combination of the two eigenvectors [26], and the contrast-response function *F*(*x*) can be measured as shown in Figure 1C and 1D. It is important to note, however, that the STC analysis does not presume the validity of the energy model [30]; conversely, the results of the analysis (Figure 1B and 1D) do not imply that the energy model provides a complete description of complex-cell responses, as shown below.

### Feature Sensitivity of Complex Cells in Response to Natural and Random Stimuli

**Figure 2:**
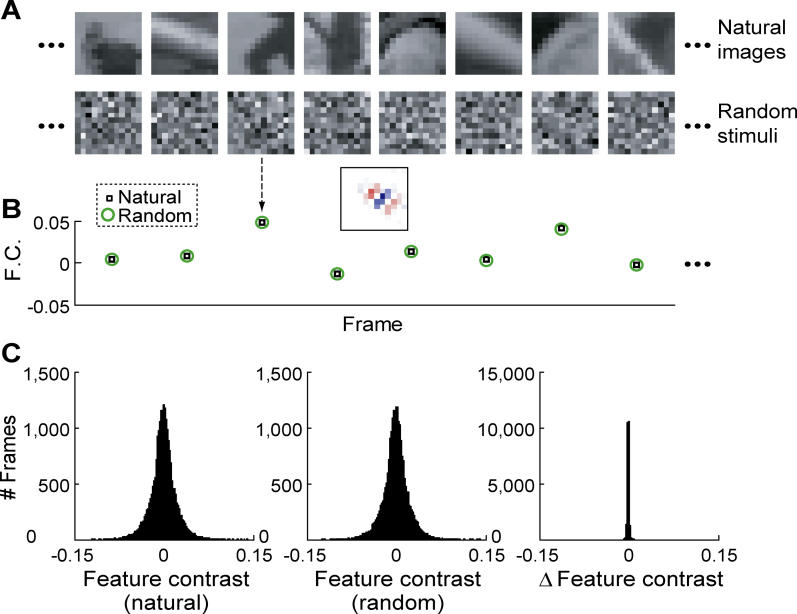
Matching of Feature Contrasts in Natural and Random Ensembles (A) Example images in the natural (upper row) and the random (lower row) ensembles, which were matched frame by frame for both global and feature contrasts. (B) Contrasts of a preferred feature of a complex cell (inset at center) in each frame of the natural (squares) and random (circles) ensembles in (A). F.C. denotes feature contrast. (C) Distributions of feature contrasts in the natural (left) and random (middle) ensembles, and the distribution of the difference in feature contrast between the two ensembles (right).

According to the energy model, the response of a complex cell to each image is completely determined by the contrasts of the preferred features in the image, *k* ^φ^•*S* and *k* ^φ+90°^•*S*. However, numerous studies have shown that many visual stimuli that are ineffective in driving a cortical neuron on their own (e.g., stimuli at non-preferred orientations or in the nonclassical RF) may strongly modulate the responses to the effective stimuli [22,31,32] and affect the neuronal sensitivity to the preferred features [26,30]. To test whether cortical feature sensitivity depends on the statistics of the images that contain both the preferred and non-preferred features, we measured the responses of complex cells to natural images and to spatially unstructured random stimuli (Figure 2A). To control for the confounding effect of contrast adaptation [33] on cortical feature sensitivity, we constructed the random-stimulus ensemble specifically for each cell such that it matched the natural-image ensemble, frame by frame, in both the global (root-mean-square) contrast and the contrasts of one or both of the preferred features of the cell (Figure 2). This was achieved by first creating an orthonormal basis set that included the significant eigenvector(s) of the cell. Then, for each natural image, a random stimulus was generated by selecting the coefficients of the basis functions such that the global and feature contrasts were matched between the natural and random stimuli (see Materials and Methods). Because of the randomness in generating most of the basis functions and in selecting their coefficients, these stimuli exhibit no clear spatial structure (Figure 2A).

**Figure 3:**
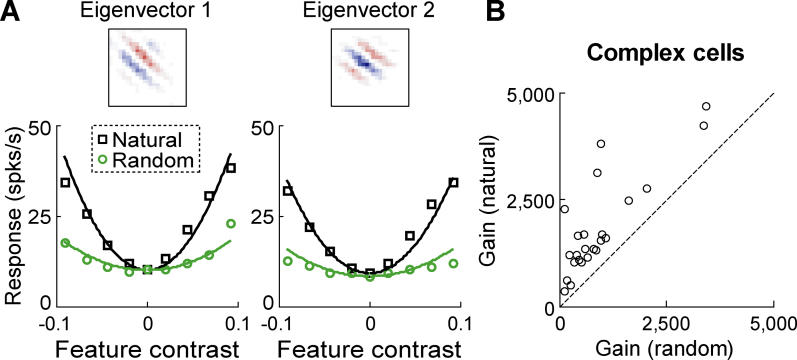
Feature Sensitivity of Complex Cells in Response to Natural Images and Random Stimuli (A) Contrast-response functions for both preferred features (insets above) of a complex cell. Curves: fits of data with quadratic functions. (B) Gain of contrast-response function (in spikes/s per unit feature contrast) for natural ensemble versus that for contrast-matched random ensemble. For this population of cells, the gain was significantly higher for the natural than for the random ensemble (*n* = 24, from 14 cells; *p* < 10^−4^, Wilcoxon signed rank test).



When we examined the contrast-response functions that were computed, for each significant eigenvector, from the responses to the natural- and random-stimulus ensembles, we found that, for both ensembles, the neuronal response increased with the magnitude of the feature contrast independently of its sign (Figure 3A), consistent with the polarity invariance of complex cells [19,20]. Interestingly, however, the amplitude of the contrast-response function was markedly higher for natural images than for random stimuli. To compare these functions quantitatively, we fit each function with

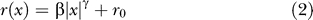



where *r* is the neuronal response, *x* is feature contrast, *γ* = 2 (based on the standard energy model of complex-cell RFs [21]; see above), and *r*_0_ and β are free parameters (Figure 3A, curves). For all the cells tested in this experiment (14 cells, 24 significant eigenvectors), we found that the gain of the contrast-response function (β), which directly reflects the feature sensitivity of the neuron, was higher in the responses to natural images (*p* < 10^−4^, Wilcoxon signed rank test; Figure 3B). This result was independent of the value of *γ* between one and three, and it remained the same if the positive and negative sides of each function were fitted separately.

In the above experiment, ten of the 14 complex cells had two significant eigenvectors. For four of these ten cells, the natural and random stimuli were matched for the feature contrasts of both eigenvectors simultaneously. For the remaining six cells, however, a random-stimulus ensemble was generated to match the natural ensemble for each eigenvector separately. In this case, it is possible that the contrasts of the two significant eigenvectors were more correlated in natural images than in the random stimuli [34], and the response of the cell to the matched eigenvector may be enhanced by the correlated presence of the other eigenvector. To test this possibility, we re-computed the contrast-response function for each significant eigenvector of the ten cells using only the frames in which the contrast of the other “un-matched” eigenvector was very low (<0.005), so that excitation of the cell due to this un-matched feature was negligible (Figure 3A). We found that the feature sensitivity was still significantly higher for the natural than for the random stimuli (*p* < 0.002, Wilcoxon signed rank test). Furthermore, if we consider only the four cells for which the contrasts of both significant eigenvectors were matched simultaneously (thus any correlation between them in natural images would be equally present in the random stimuli), the feature sensitivity was still significantly higher for the natural than for the random stimuli (*p* < 0.01). Thus, the higher feature sensitivity of the complex cells in response to natural images is not due to any correlation between the two significant eigenvectors.

Another potential problem in the above experiment is that the preferred features were estimated from the responses to natural images, the non-Gaussian statistics of which may cause bias in the estimation of the preferred features with STC. In addition, although for most of the cells (12/14) the preferred features and their contrast-response functions were computed from the responses to different repeats of the natural-image ensemble, it is possible that using the same stimuli for both computations can introduce bias in the measured sensitivity. To control for these potential biases, for a separate set of complex cells we replaced the features estimated with natural images (see Figure 1B) with Gabor functions whose parameters were chosen based on the orientation and spatial-frequency tuning of the neuron measured with sinusoidal gratings. These Gabor functions resembled the significant eigenvectors measured with natural images (Figure 1B). When we compared the responses to a natural-image ensemble and a random-stimulus ensemble matched for both the global contrast and the contrast of the Gabor function (analogous to the analyses shown in Figures 2 and 3), the neuronal sensitivity to these Gabor functions was also found to be higher in the responses to natural images (*n* = 10, from ten cells; *p* < 0.05). This indicates that the effect is robust with respect to small variations in the spatial structure of the preferred visual features, and it is not due to the potential biases in feature estimation with natural images. Such an effect is not predicted by the existing models of complex-cell RFs [21,34,35], and it indicates that cortical feature sensitivity depends strongly on the image statistics.

### Feature Sensitivity of Simple Cells

**Figure 4:**
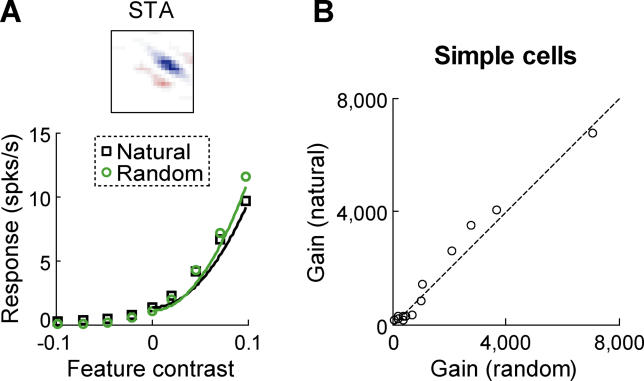
Feature Sensitivity of Simple Cells in Response to Natural Images and Random Stimuli (A) Contrast-response function for the preferred feature (inset above) of a simple cell. Curves: fits of data with quadratic functions (for positive feature contrasts only). (B) Gain of contrast-response function, as in Figure 3B (*n* = 14, from 14 cells).



Surprisingly, for simple cells we found no difference in feature sensitivity between their responses to natural and random stimuli. The preferred feature of each simple cell (Figure 4A, inset), which directly corresponds to the classical RF of the cell, was estimated with a modified spike-triggered average (STA) analysis that corrects for the spatial correlations in natural images [23,36] (see Materials and Methods). We then constructed a random-stimulus ensemble that was matched to a natural-image ensemble, frame by frame, for both the global contrast and the contrast of the preferred feature (similar to the method for complex cells; see Figure 2). Responses to these contrast-matched natural and random ensembles were recorded, and the contrast-response functions were computed. Not surprisingly, the contrast-response functions measured with both ensembles were monotonic (see Figure 4A), consistent with the polarity sensitivity of simple cells [19]. To obtain a quantitative measure of the simple-cell feature sensitivity, we fit the positive side of each contrast-response function with

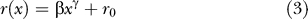



where *r* is the neuronal response, *x* is the feature contrast, *γ* = 2, and *r*_0_ and β are free parameters. Across the population of simple cells studied, the contrast-response gain (β) was not significantly different between the natural images and the random stimuli (*n* = 14, from 14 cells; *p* > 0.55) (Figure 4B). To compare the results between simple and complex cells directly, for each cell we computed Δβ = β_natural_ − β_random_ (β_natural_ and β_random_ are contrast-response gains for natural and random stimuli, respectively; for cells with two significant eigenvectors, Δβ was averaged between the two). We found that Δβ is significantly higher for complex cells than for simple cells (*p* < 0.001, Wilcoxon rank sum test).

**Figure 5:**
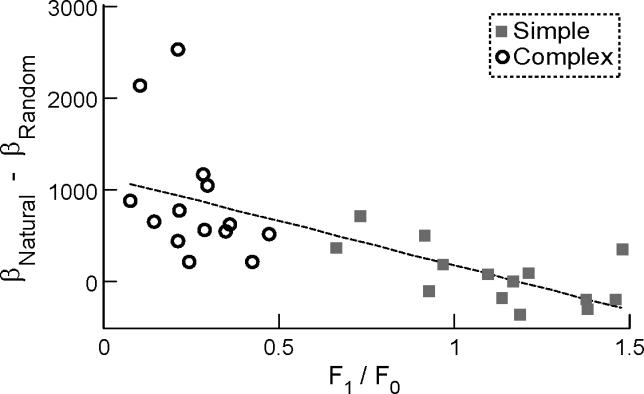
Difference in Feature Sensitivity between the Responses to Natural and Random Stimuli as a Function of *F*_1_ */F*_0_ Each symbol represents data from one cell. For complex cells with two significant eigenvectors, the sensitivity difference was averaged between the two eigenvectors. Dashed line: linear fit.

Note that, in this study, a cell was classified as simple if *F*_1_ */F*_0_ > 0.6 (see Materials and Methods). This criterion is somewhat arbitrary, as V1 neurons may lie on a simple-complex continuum rather than belong to one of two distinct categories [37–39]. To test whether our classification criterion affects the observed difference in feature sensitivity between simple and complex cells, we plotted Δβ against *F*_1_/*F*_0_ for each cell (Figure 5). While the distributions of both Δβ and *F*_1_/*F*_0_ may be continuous, there is a significant negative correlation between Δβ and *F*_1_/*F*_0_ (*p* < 10^−4^), and the cells with the highest *F*_1_/*F*_0_ (most “simple-cell like”) tend to exhibit the lowest Δβ. If we use the standard criterion [40] and classify the five cells with 0.6 < *F*_1_/*F*_0_ < 1 as complex cells (thus using the first eigenvector of their STC instead of STA as the preferred feature), Δβ is still significant for complex cells (*p* < 5 × 10^−4^, Wilcoxon signed rank test) but not for simple cells (*p* > 0.4), and Δβ is significantly different between simple and complex cells (*p* < 10^−4^, Wilcoxon rank sum test). Thus, the observed difference in feature sensitivity between simple and complex cells is not sensitive to the criterion used for simple/complex classification. Additional analyses have demonstrated that this difference is also not due to the different methods (STA versus STC) used to identify the preferred visual features (Protocol S1; Figure S1).

### Detectability of Preferred Features from Complex-Cell Responses

**Figure 6:**
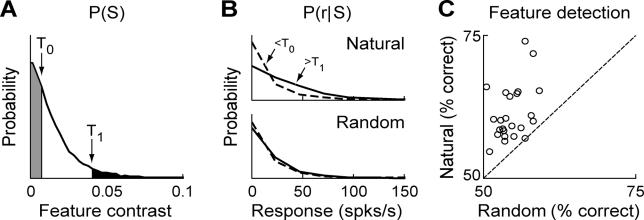
Detectability of Features from Neuronal Responses to Natural Images and Random Stimuli (A) Probability distribution of feature contrast in a natural ensemble (or, equivalently, its matched random ensemble). For simplicity, only the positive side (feature contrast >0) is shown. Gray shading: feature contrasts near zero (<*T*_0_, here *T*_0_ *=* 0.007, “feature absent”); black shading: high feature contrasts (>*T*_1_, here *T*_1_ = 0.04, “feature present”). (B) Conditional probability distributions of responses evoked by natural images (upper) and random stimuli (lower). Solid lines: response distributions when the feature was present in stimulus (black shading in [A]); dashed lines: distributions when the feature was absent (gray shading in [A]). (C) Feature detectability in natural images versus that in matched random stimuli, for the same population of cells shown in Figure 3B. Detectability was measured as the percentage of trials in which stimuli were correctly classified as “feature present” or “feature absent” (see Materials and Methods).

Functionally, the observed difference in complex-cell feature sensitivity predicts that the preferred features are more detectable in natural images than in random stimuli, but this difference could be eliminated if the increased sensitivity is accompanied by a similar increase in response noise. We thus tested this prediction directly using signal detection theory [41]. For simplicity, we defined two sets of stimuli in each ensemble: those with high feature contrast (>*T*_1_; Figure 6A, black shading, referred to as “feature present”) and those with near-zero feature contrast (<*T*_0_; gray shading, “feature absent”). Detectability of the feature was measured by how reliably the “feature-present” stimuli could be distinguished from the “feature-absent” stimuli based on the neuronal response (see Materials and Methods). Figure 6B shows the probability distribution of the neuronal response when a feature was either present (solid line) or absent (dashed line), for both the natural- and random-stimulus ensembles. For the natural ensemble, the neuron was more likely to fire at higher rates when the feature was present than when it was absent, as expected. Such a difference in the response probability allows correct classification of the two sets of stimuli at a level well above chance (50%; see Figure 6C).

For the random ensemble, however, the two response distributions were much less distinguishable (Figure 6B), resulting in a lower percentage of correct classifications. This result is inconsistent with the energy model, which would predict that the upper and lower plots in Figure 6B would be identical. For the population of cells studied, detectability of the preferred features from the neuronal responses was significantly higher for the natural-stimulus ensemble (*n* = 24, *p* < 10^−4^, Wilcoxon signed rank test; Figure 6C), and this result was independent of the criteria used to select the two stimulus sets (Figure 6A, *T*_0_ and *T*_1_).

### Dependence of Cortical Feature Sensitivity on Power and Phase Spectra

**Figure 7:**
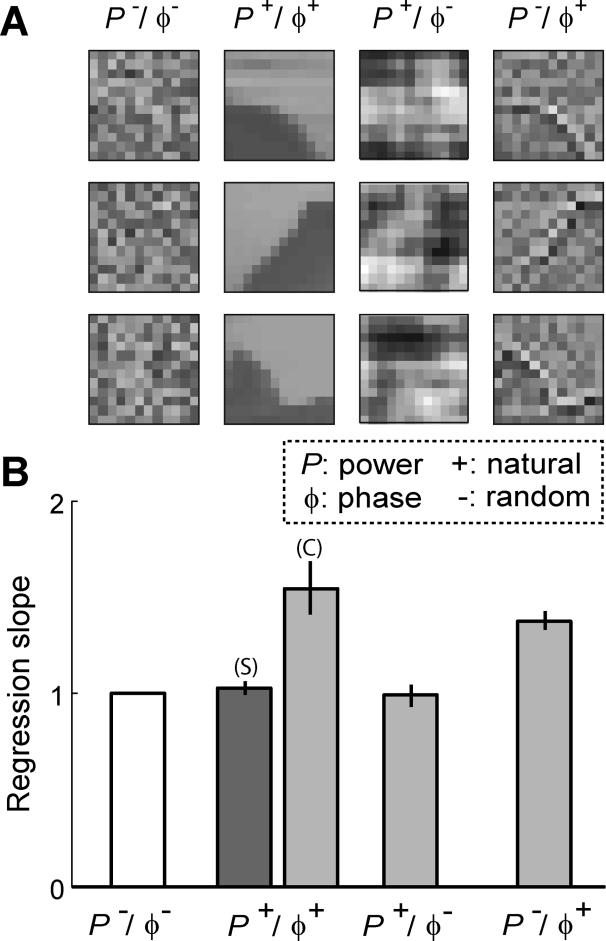
Effects of Power and Phase Spectra of Stimuli on Cortical Feature Sensitivity (A) Four classes of stimulus ensembles with distinct combinations of power (*P*) and phase (φ) characteristics; +: natural; −: random. Example stimuli from each class are shown. The *P^−^*/φ*^−^* and *P^−^*/φ^+^ stimuli are matched for both the global contrast and the feature contrasts for a particular complex cell. (B) Summary of cortical feature sensitivity (contrast-response gain; see Figure 3B) for the stimulus classes in (A). In each experiment, a random (*P^−^*/φ*^−^*) stimulus ensemble was generated to match *P* ^+^/φ^+^, *P* ^+^/φ*^−^*, or *P^−^*/φ^+^ in global and feature contrasts (see Figure 2 and Materials and Methods), and the measured contrast-response gain was plotted against the gain for *P^−^*/φ*^−^* (as in Figure 3B). Bar represents slope of linear regression (through origin); >1 indicates higher contrast-response gain relative to *P^−^*/φ*^−^*. Error bar: ± standard deviation. *P* ^+^/φ^+^ bars for simple (S) and complex (C) cells were computed from data in Figures 3B and 4B, respectively, and *P* ^+^/φ*^−^* (*n* = 10, from six cells) and *P^−^*/φ^+^ (*n* = 11, from six cells) were from largely nonoverlapping populations of complex cells (one cell was used in two separate experiments).

What stimulus property is responsible for the higher feature sensitivity of complex cells in response to natural rather than to random stimuli? Since the natural- and random-stimulus ensembles were matched for both global and feature contrasts (see Figure 2), the difference in contrast-response gain (see Figure 3) cannot be attributed to cortical contrast adaptation [33]. Instead, it is likely due to the differences in the spatial characteristics of the stimuli. For natural images, power (*P*) decreases with spatial frequency [1,2], and nearby frequencies tend to have similar phases (φ) [5], due to the prevalence of surfaces and edges of objects. White noise, on the other hand, has a flat power spectrum and random phase structure. For convenience, we use *P* ^+^/φ^+^ and *P^−^*/φ*^−^* to represent the statistical properties of natural and white-noise stimuli, respectively (where “+” represents natural). In order to distinguish the effects of power and phase spectra on cortical feature sensitivity (Figure 3), we manipulated each property separately, yielding two classes of synthetic stimulus ensembles: the “natural-power” ensemble, in which each image had a natural power but a random phase spectrum (*P* ^+^/φ*^−^*), and the “natural-phase” ensemble, in which each image had a random power but a natural phase spectrum (*P^−^*/φ^+^) (Figure 7A; see Materials and Methods).

Visually, natural-power stimuli tend to exhibit smooth luminance variations in space (typical of natural images but not of white noise), but they appear amorphous owing to the lack of well-defined edges and contours. Natural-phase stimuli, however, contain reduced low-frequency signals but retain and enhance the edges in natural images. Thus, these synthetic stimuli capture complementary spatial characteristics of natural images.

When we compared the feature sensitivity of each complex cell in response to each of these ensembles with that to a random ensemble (matched for global contrast and the feature contrasts for all significant eigenvectors; see Materials and Methods), we found that the contrast-response gain was significantly higher for *P^−^*/φ^+^ (*n* = 11, from seven cells; *p* < 0.02, Wilcoxon signed rank test) but not for *P* ^+^/φ*^−^* (*n* = 10, from six cells; *p* > 0.15) (Figure 7B). This indicates that the increased feature sensitivity is due not to the power spectrum of natural images, but to their spatial-phase regularities.

## Discussion

The main finding of this study is a new response property of complex cells pertaining to the coding of natural stimuli. Although the hypothesis of efficient coding by cortical neurons in terms of redundancy reduction among a population of neurons has been examined in theoretical studies [2,4,9,10,14–18], there have been relatively few experimental studies on the relationship between cortical responses and the statistical properties of natural stimuli [24,42,43]. In the present study, we have shown that the response sensitivity of complex cells to their preferred features is higher for natural images than for random stimuli (see Figure 3), leading to increased feature detectability in the cortical representation of natural stimuli (see Figure 6).

Since these stimulus ensembles were matched frame by frame for the contrasts of the significant eigenvectors (*k* ^φ^•*S* and *k* ^φ+90°^•*S;* see Figure 2), they should activate the energy-model mechanism to the same extent. The observed difference in cortical responses can therefore be attributed to the numerous other features that were not matched between the two ensembles (see Materials and Methods). Previous studies have shown that the non-preferred stimuli can affect the gain of the neuronal contrast-response function for the preferred stimuli [26], and these effects (which we refer to as “contextual modulation”) have been modeled as divisive normalization [34,35]. This normalization model can be described as



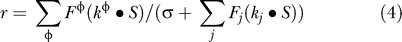



where ∑_φ_ *F* ^φ^(*k* ^φ^•*S*) corresponds to the energy model, *k_j_* and *F_j_* are the RF and contrast-response function of the *j*th divisive subunit, respectively, and *σ* is a constant. This model can account for a range of nonlinear cortical-response properties, including contrast-gain control and cross-orientation inhibition [44]. Our finding suggests that the non-preferred features (*k_j_*) provide different degrees of suppressive modulation of the responses to the natural and random stimuli, resulting in higher feature sensitivity for natural images. Note that while gain control and contextual modulation may enhance the efficiency of visual coding by optimizing information transmission in individual neurons [45], reducing inter-neuronal correlations [34,42], or increasing response sparseness [42,46], a potential detrimental effect is a reduction in the sensitivity of cortical neurons to their preferred features when the features are embedded in complex stimuli. Our results suggest that this mechanism is tuned to the spatial statistics of natural images so as to reduce the suppression of the responses to the preferred features.

A well-known form of suppressive modulation is cross-orientation inhibition [32,47], and the higher feature sensitivity of complex cells in response to natural images could be caused by fewer cross-oriented components in these stimuli. To test for this possibility, we estimated the contrast energy at the orientation orthogonal to the preferred features as the mean-square contrast of the preferred features rotated by 90°. We found no significant difference in this contrast energy between the natural- and the random-stimulus ensembles (*p* > 0.4). In addition to the total energy over the entire ensemble, we also examined whether, in each stimulus frame, the contrast energy of the preferred features is correlated with the energy of the cross-oriented components. We found that such correlation is negligible (correlation coefficient within ±0.01) in both the natural and random ensembles. These results suggest that the observed difference in the feature sensitivity of complex cells in response to the natural and random stimuli is not likely to be due to the difference in cross-orientation inhibition. We also considered the possibility that the natural images contain less global contrast (and thus less non-preferred feature contrast) than the random stimuli within the RF of the cell, since matching the stimuli for global contrast over the entire image does not guarantee a precise match within the RF region. However, further analyses suggested that this could not account for the observed difference in feature sensitivity (Protocol S2).

Aside from suppressive modulation, there may be additional excitatory visual features (*k* ^φ^) not identified by our STC analysis, a possibility indicated by a recent study in macaque V1 [30]. These additional excitatory features may be more correlated with the identified features in natural images than in random stimuli, resulting in higher response gains for the identified features. If this were the case, it would suggest that the improved feature sensitivity of complex cells is mediated by tuning of the additional excitatory features to the statistics of natural images. Note that, in the traditional view, the function of complex cells in detecting oriented edges or lines is primarily mediated by a pair of Gabor filters described by the energy model. Our current finding, together with that in macaque V1 [30], indicates that these cells have more specialized RF properties. They can carry out more refined feature detection by responding more vigorously to the oriented edges and contours that are meaningful features in natural images (e.g., those belonging to the borders of physical objects) than to random stimuli that contain the same contrasts of the pair of Gabor filters.

Interestingly, while this property is highly robust in complex cells (see Figure 3), it is virtually absent in simple cells (see Figures 4 and 5). This difference may be due to the differential laminar distributions of the two cell types and the different neuronal circuitry contributing to their contextual modulation. Related to our finding, an earlier study showed that the repulsive shift in cortical orientation tuning induced by surround visual stimulation, which may increase the efficiency of visual coding, is found only for complex cells and not for simple cells [48].

A study of feature detection in human vision led to the suggestion that complex cells act as detectors for phase congruence in visual images [49]. Recent physiological experiments have shown that the responses of complex cells to compound gratings depend on the relative spatial phase of the gratings [50]—an effect not accounted for by the energy model. In the present study, the higher feature sensitivity to natural images is also due to their nonrandom phase structure rather than to their power spectra (see Figure 7). This indicates that the phase regularity of natural images, due in large part to the prevalence of well-defined edges and contours [5,49], can strongly affect the cortical-response gain. This effect and that observed with compound gratings [50] may share common mechanisms. At the perceptual level, although the non-flat power spectrum is a well-studied, robust feature of natural scenes [1,2], the phase spectrum in fact carries most of the information that allows the animal to distinguish one scene from another [51–53]. Thus, along the mammalian visual pathway, coding of natural scenes appears to be refined at multiple stages: While the RF structure of retinal and thalamic neurons is adapted to the power spectra of natural stimuli to reduce coding redundancy [3,11–13], and the RFs of cortical simple cells are adapted to the phase structure of natural stimuli to attain sparse coding [14–16], gain control of feature sensitivity of complex cells is tuned to the phase regularity of natural scenes to improve the saliency of relevant visual features.

## Materials and Methods

### Overview of experimental paradigm

In this study, each cortical neuron was subjected to a sequence of inter-dependent experiments and analyses. To facilitate understanding of this experimental design, we provide in this section a brief outline of the major steps involved in studying each cell (details of these steps are provided in subsequent sections).

In Step 1, we estimated the preferred feature(s) of the cell from the recorded responses to an ensemble of natural images (see Figure 1A), using STA for simple cells and STC for complex cells.

In Step 2, we created a random-stimulus ensemble that was matched to a “target ensemble” (which was a natural-image, a natural-phase, or a natural-power ensemble; see Visual stimulation) for both global and feature contrasts (see, for example, Figure 2). Note that this step depends on the outcome of Step 1, since matching the feature contrast requires knowing the precise structure of the preferred feature(s).

In Step 3, we recorded the responses of the neuron to both the target and the random ensembles (created in Step 2) and computed the contrast-response functions for each feature from both responses (see Figure 3A).

In Step 4, we compared the gains (*β*) of the contrast-response functions computed from the responses to the target and random ensembles (see Figures 3B, 4B, and 7B).

In one control experiment, we replaced the preferred features estimated in Step 1 with Gabor functions whose parameters were chosen based on the responses of the cell to drifting gratings. Steps 2–4 then followed as above.

### Recording

Animal-use procedures were as previously described [26] and approved by the Animal Care and Use Committee at the University of California, Berkeley. A total of 18 cats (weighing 2–6.5 kg each) were used. Single-unit recordings were made in area 17 using tungsten electrodes (A-M Systems, http://www.a-msystems.com). Cells were sampled at all laminar locations. Unit isolation was based on cluster analysis of waveforms. Cells were excluded if their mean firing rates were <1 spike/s or if their response correlations between repeats were at chance level. The firing rates of the cells included in this study ranged from 1 to 76 spikes/s (median: 6 spikes/s). Cells were classified as simple or complex based on the ratio of the first harmonic (*F*_1_) and the mean (*F*_0_) of the response to a drifting grating stimulus [40] (simple cell if *F*_1_ */F*_0_ > 0.6; this criterion was used because for all the cells in our sample with 0.6 < *F*_1_ */F*_0_ < 1, RFs measured by STA exhibited clear spatial structure).

### Visual stimulation

Visual stimuli were generated with a PC and presented with a Barco monitor (size 40 × 30 cm, refresh rate 120 Hz, maximum luminance 80 cd/m^2^). Luminance nonlinearities were corrected through software. Four classes of stimulus ensembles were used in this study: natural-image, natural-power, natural-phase, and random-stimulus ensembles.

### Natural-image ensemble (*P* ^+^/φ^+^)

Raw images were selected at random from a database consisting of a variety of digitized natural movies [16], and the center patch (12 × 12 pixels) of each image was retained. To maximize the diversity of images, we measured the similarity between each pair of stimuli *S_i_* and *S_j_* in the ensemble by their dot product



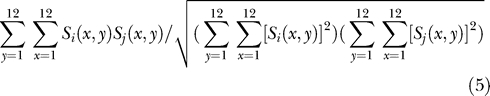



For similar images (dot product > 0.95), either *S_i_* or *S_j_* was excluded. Three distinct natural ensembles were used in this study (with no common image between ensembles). Unlike natural movies, these images were presented in a random sequence; the absence of temporal correlation greatly facilitated the STC analysis [27] (see below). However, since natural images are highly variable in their global contrast (measured by root-mean-square contrast



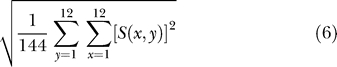



where −1 ≤ *S*(*x, y*) ≤ 1, and 1 and −1 represent highest and lowest luminance of the monitor, respectively), such stimuli may invoke contrast adaptation [33] that could confound the interpretation of the results. To control for this problem, we scaled each image such that all frames had the same global contrast (0.32). Any frame that could not be scaled without violating −1 ≤ *S*(*x, y*) ≤ 1 was excluded from the ensemble.

### Natural-power ensemble (*P* ^+^/φ^−^)

First, we computed the Fourier transform of every image in a natural ensemble (*P* ^+^/φ^+^). We then generated each image in the *P* ^+^/φ^−^ ensemble as



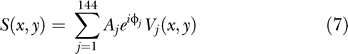



where *V_j_* is the  *j*th Fourier component of the corresponding natural image, *A_j_* is the amplitude spectrum of the natural image, and the phase spectrum (φ*_j_*) was obtained from a randomly generated 12 × 12 image, in which the luminance value of each pixel was drawn randomly from a uniform distribution on [0, 1]. The resulting image had the same power spectrum as its corresponding natural image, but the spatial phase spectrum is random. The global contrast of each image was the same as that for the natural images (0.32).

### Natural-phase ensemble (*P* ^−^/φ^+^)

For each image in the natural ensemble (*P* ^+^/φ^+^), we retained its phase spectrum, but drew the amplitude at each frequency randomly from a uniform distribution on [0, 1], yielding the corresponding image in the natural-phase ensemble. These natural-phase images largely preserve the edges in natural images, but the spatial power spectrum of each image is random; the average power spectrum over the entire ensemble is flat.

### Random-stimulus ensemble (*P* ^−^/φ^−^)

We generated the random ensemble specifically for each cell and each target ensemble (which could be a natural-image, natural-power, or natural-phase ensemble) to be matched for both the global and feature contrasts. First, we created an orthonormal basis set (*V_i_,* where *i* = 1, 2, …, 144) that included the preferred feature(s) of the cell (i.e., for a complex cell with two significant eigenvectors that are matched simultaneously, *V*_1_ and *V*_2_ represent these eigenvectors, and *V*_3_, … *V*_144_ are arbitrary aside from the requirement of orthonormality; for a simple cell, *V*_1_ represents its preferred feature measured by STA, and *V*_2_, … *V*_144_ are arbitrary). This was achieved in the following steps.

First, we generated a 144 × 144 symmetric matrix from the two significant eigenvectors (*k* ^φ^ and *k* ^φ+90°^) as



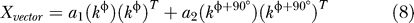



where *a*_1_ and *a*_2_ were large but unequal numbers (e.g., *a*_1_ = 10^9^, *a*_2_ = 10^8^).

Second, we generated another 144 × 144 symmetric matrix using random vectors (*U_i_,* where *i* = 1, 2, …, *n,* and where *n* >> 144) as



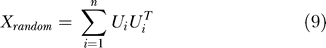



with each component of *U_i_* drawn randomly from a normal distribution (mean = 0, variance = 1).

Third, we calculated the eigenvectors of *X_vector_* + *X_random_* (*V_i_,* where *i* = 1, 2, …, 144), and these eigenvectors were used as the basis set for generating the random stimuli. Note that the large coefficients *a*_1_ and *a*_2_ ensure that the two preferred features (*k* ^φ^ and *k* ^φ+90°^) can be arbitrarily close to the first two eigenvectors (*V*_1_ and *V*_2_).

Then, for each image in the target ensemble, we generated a corresponding “random” stimulus as



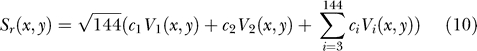



The coefficients *c_i_* were selected such that (1) *c*_1_ and *c*_2_ were the contrasts of the first and second preferred features in the target image, which ensured that the random stimulus was matched to the target image for feature contrast (see Figure 2), and (2) *c_i_,*where *i* = 3, 4, …, 144 were drawn randomly from a normal distribution and then scaled so that



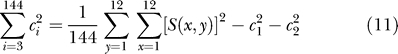



where *S* is the target image. This ensured that the global stimulus contrast (equal to



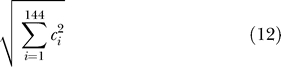



for an orthonormal basis set) was also matched to that of the target image. If any pixel of *S_r_*(*x, y*) fell outside the range [*−*1, 1], this frame was discarded and new *c_i_* were selected, until −1 ≤ *S _r_*(*x, y*) ≤ 1. This process was repeated for all the frames in the target ensemble. Note that although the stimuli that satisfied constraints (1) and (2) are, strictly speaking, not random, they exhibit no clear spatial structure (Figure 2A) because of the randomness in choosing most of the basis functions (*V*_3_, …, *V*_144_) and their coefficients (*c*_3_, …, *c*_144_). The spatial power spectra of these stimuli are flat, as for white noise.

All the stimuli described above were updated every five frames, corresponding to an effective frame rate of 24 Hz. Each ensemble consisted of 24,000 effective frames (referred to in the main text simply as “frames”) and was 16.7 min long. For each cell, the size of the images was adjusted to be slightly larger than the classical RF, although across the population of cells the relationship between the RF and the stimulus patch varied somewhat with respect to location and size. In experiments comparing feature sensitivity, we interleaved the presentations of target and random ensembles in order to control for the effects of the slow drift in the physiological state of the animal.

### STC analysis

This technique has been used in previous studies to analyze the nonlinear-response properties of sensory neurons [25] and has been shown to be effective for computing the preferred features of V1 complex cells [26,27,30]. For all the cells in this study (except those used to test Gabor functions), we estimated the preferred features using natural images (12 × 12 pixels). Since natural images contain significant spatial correlations, it was necessary to modify the STC analysis in order to compute the preferred features. Details of this method can be found in Touryan et al. [27]. Briefly, we first corrected for the correlations in the stimuli by “whitening” each image in the ensemble:



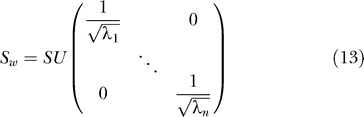



where *S* is a vector representation of the stimulus (luminance in each pixel at each frame), *U* is a matrix containing the eigenvectors of the covariance matrix of *S,* and *λ*_1*, …, n*_ are the corresponding eigenvalues. As a result, *S_w_* represents the stimulus in the whitened space. STC analysis for white-noise stimuli [25,26] was then applied to the ensemble *S_w_* to identify significant eigenvectors (*V_sig_*). The preferred features are then computed as



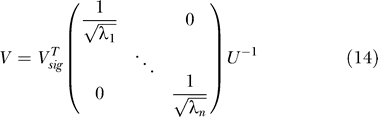



Since some eigenvalues (*λ*) for natural stimuli are very small, the “whitening” step will result in noise amplification. To solve this problem, a cutoff threshold was chosen such that whitening is performed only for eigenvectors above this cutoff (in this study, 50 out of 144 eigenvectors [27]).

### STA analysis

This technique has been used previously to estimate the linear RFs of sensory neurons [23,36]. Briefly, to analyze the responses to white-noise stimuli, *V_STA_* is calculated as the average of the spike-triggered stimulus ensemble. To analyze the responses to natural stimuli, the stimulus correlations are corrected for by normalizing *V_STA_* by the covariance matrix of *S* (see STC analysis). To avoid noise amplification, this normalization was performed only above the cutoff (50/144), identical to that used for the modified STC analysis.

Although both STA and STC can be used to estimate preferred spatiotemporal features, in the present study we focused on the space domain in order to improve the signal-to-noise ratio of the estimate. In preliminary studies, we computed the spatiotemporal features of a subset of cells and found that the signals in the features are almost completely contained in the spatial feature at a delay of one frame (Figure S2). Thus in the present study the analysis was performed only at that delay. For each cell, we used the responses to 1–3 repeats of a natural-image ensemble to compute the preferred features.

### Contrast-response function

The contrast of preferred feature *k* ^φ^ (*x, y*) in stimulus *S*(*x, y*) is measured as the dot product of *k* ^φ^ and *S*:



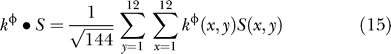



(see Figure 1C); *k* ^φ^ satisfies



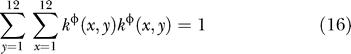



Since −1 ≤ *S*(*x, y*) ≤ 1, where 1 and −1 represent the highest and lowest luminance of the monitor, respectively, the above definition ensures that the feature contrast in each stimulus is bound between *−*1 and 1 (although, in practice, these limits were never reached with the stimulus ensembles used in this study). For each feature, the contrast-response function was measured from the neuronal responses to 1–3 repeats of an ensemble; for nearly all cells (38/40), these repeats were distinct from those used to estimate the preferred features, in order to avoid bias [29].

### Analysis of feature detectability

For each visual feature and each stimulus ensemble, 50,000 trials were performed in silico for both the positive and negative values of the feature contrast. In each trial, a pair of neuronal responses (*r_p_* and *r_a_*), corresponding to “feature-present” and “feature-absent” stimuli respectively, were generated based on the probability distributions shown in Figure 6B (solid and dashed lines). The larger response of the pair was classified as the response to the “feature-present” stimulus (*r_p_*′); trials in which the responses were equal were excluded. The percentage of trials with correct classification (*r_p_*′ matches *r_p_*) was computed for both the positive and negative feature contrasts, and the results were averaged. The result shown in Figure 6C was qualitatively independent of the specific values of *T*_0_ and *T*_1_ over a wide range.

## Supporting Information

Figure S1 Comparison between STA and Significant Eigenvector of STC for Simple Cells Results from two cells are shown. Upper, STA. Middle, STC eigenvector. Lower, STC eigenvalues, as in Figure 1B.
(299 KB PDF).

Figure S2 Estimated Spatiotemporal Features of a Complex Cell Note that the signals in the features are almost completely contained in the spatial features at a delay of one frame.
(278 KB PDF).

Protocol S1 Analysis of Simple-Cell Feature Sensitivity Using STC (41 KB PDF).

Protocol S2 Global Contrast within RF of the Cell (19 KB PDF).

## Abbreviations

RF: receptive field
STA: spike-triggered average
STC: spike-triggered covariance
V1: primary visual cortex
