# Supplementary material for: Cortical Sensitivity to Visual Features in Natural Scenes
Source: PLoS Biol. 2005 Sep 27;3(10):e342. doi: 10.1371/journal.pbio.0030342 (PMC1233414; doi:10.1371/journal.pbio.0030342)
Supplement: Supplementary file 1 [file pbio.0030342.sd001.pdf]

Text S1. Analysis of simple cell feature sensitivity using STC.

To further ensure that the difference between simple and complex cells in feature sensitivity is not due to the different methods used to estimate the preferred features (STA vs. STC), we also analyzed the responses of all the simple cells using STC. As shown in Supplementary Figure S2, the significant eigenvector of STC is similar to STA in spatial structure (although, because they are not identical, the feature contrast based on the significant eigenvector of STC was not precisely matched between the natural and random ensembles in our experiments). We then computed the contrast-response functions for this eigenvector from the responses to both natural and random stimuli to determine β_{natural} and β_{random}. We found that $\Delta\beta$ (β_{natural} - β_{random}) for simple cells based on STC is still significantly lower than $\Delta\beta$ for complex cells (classifying simple and complex cells using $F_1/F_0 = 0.6$, $p < 0.02$; using $F_1/F_0 = 1$, $p < 0.005$). Thus, the difference in feature sensitivity between simple and complex cells is not due to the different methods for estimating the preferred visual features.
