# Supplementary material for: Cortical Sensitivity to Visual Features in Natural Scenes
Source: PLoS Biol. 2005 Sep 27;3(10):e342. doi: 10.1371/journal.pbio.0030342 (PMC1233414; doi:10.1371/journal.pbio.0030342)
Supplement: Supplementary file 2 [file pbio.0030342.sd002.pdf]

Text S2. Global contrast within RF of the cell.

Although the natural and the random stimuli were matched for global contrast over the entire image, this does not guarantee that they were matched for global contrast within the RF of the cell. If, for example, the natural images contain less global contrast (and thus less non-preferred feature contrast) than the random stimuli within the RF, there may be less suppressive modulation of the cortical responses to the preferred features. To test this possibility, we estimated the contrast energy (mean square contrast) within the RF of each cell as a weighted sum of the mean square contrast at each pixel. The weight, which represents the RF strength at each pixel [1], was set to be proportional to the Gaussian profile of the Gabor functions that approximate the significant eigenvectors of the cell (Figure 1B). We then compared the contrast energy within the RF between P^{+}/ϕ^{+} and P^{-}/ϕ^{-} or between P^{-}/ϕ^{+} and P^{-}/ϕ^{-} that were matched for feature and global contrasts. We found only slight differences in contrast energy within the RF (<10% for all cells), and no correlation was found between the mismatch in contrast energy and the observed difference in cortical feature sensitivity. For a set of cells with contrast mismatch <1% (7 cells, 11 significant eigenvectors), the feature sensitivity was still significantly higher for stimuli with the natural phase spectra than for the random stimuli ($p < 0.02$, Wilcoxon signed rank test). Thus, the higher feature sensitivity is not likely to be due to a reduction in global contrast within the RF.

Reference

1. Carandini M (2004) Receptive fields and suppressive fields in the early visual system. In: Gazzaniga MS, editor. The Cognitive Neurosciences. Cambridge, MA: MIT Press. pp. 313-326.
