# Supplementary material for: Cortical Sensitivity to Visual Features in Natural Scenes
Source: PLoS Biol. 2005 Sep 27;3(10):e342. doi: 10.1371/journal.pbio.0030342 (PMC1233414; doi:10.1371/journal.pbio.0030342)
Supplement: Supplementary file 3 [file pbio.0030342.sg001.pdf]

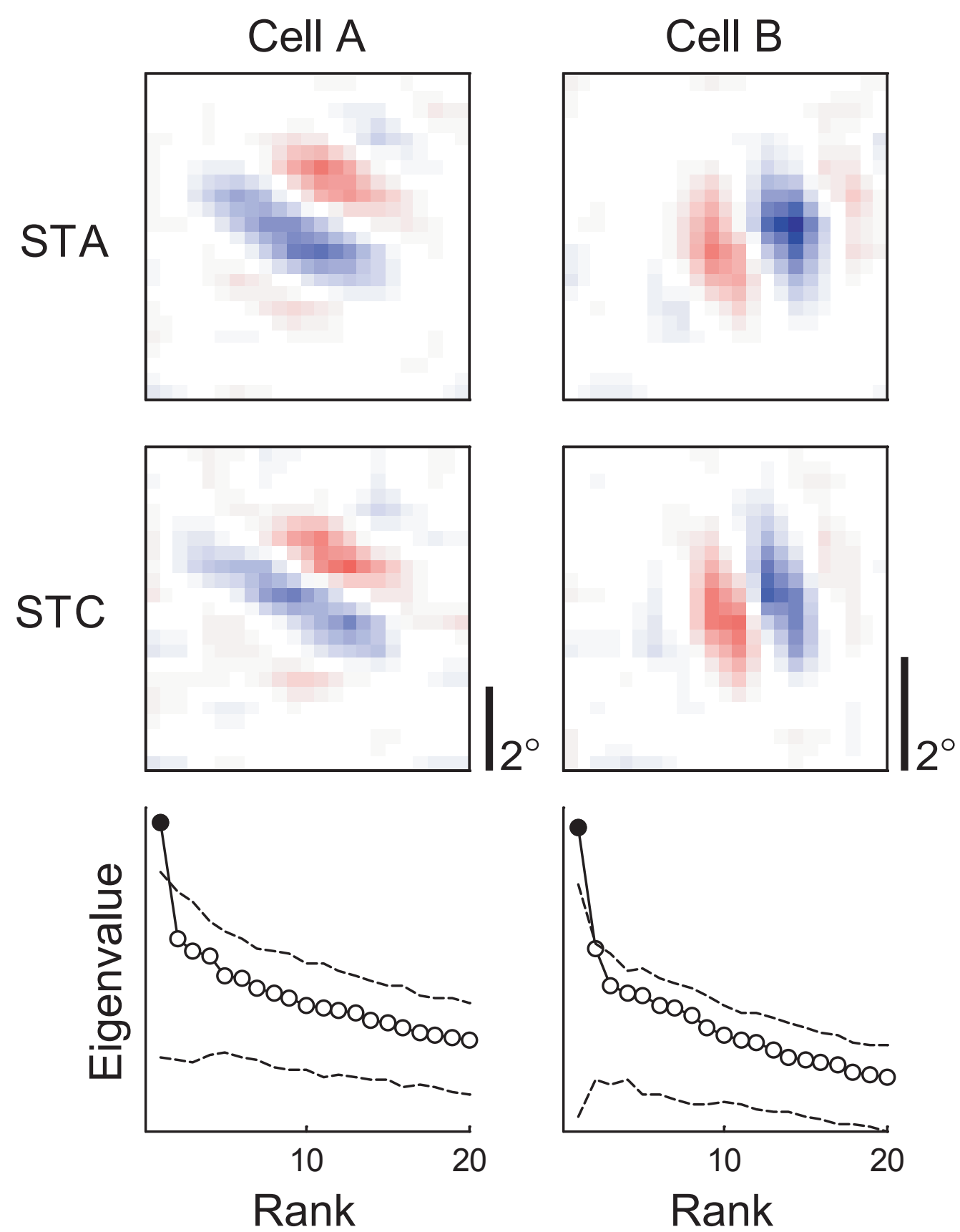

Supplementary Figure 1. Comparison between STA and significant eigenvector of STC for simple cells. Results from two cells are shown. Upper, STA. Middle, STC eigenvector. Lower, STC eigenvalues, as in Fig1B.
