# Supplementary material for: Cortical Sensitivity to Visual Features in Natural Scenes
Source: PLoS Biol. 2005 Sep 27;3(10):e342. doi: 10.1371/journal.pbio.0030342 (PMC1233414; doi:10.1371/journal.pbio.0030342)
Supplement: Supplementary file 4 [file pbio.0030342.sg002.pdf]

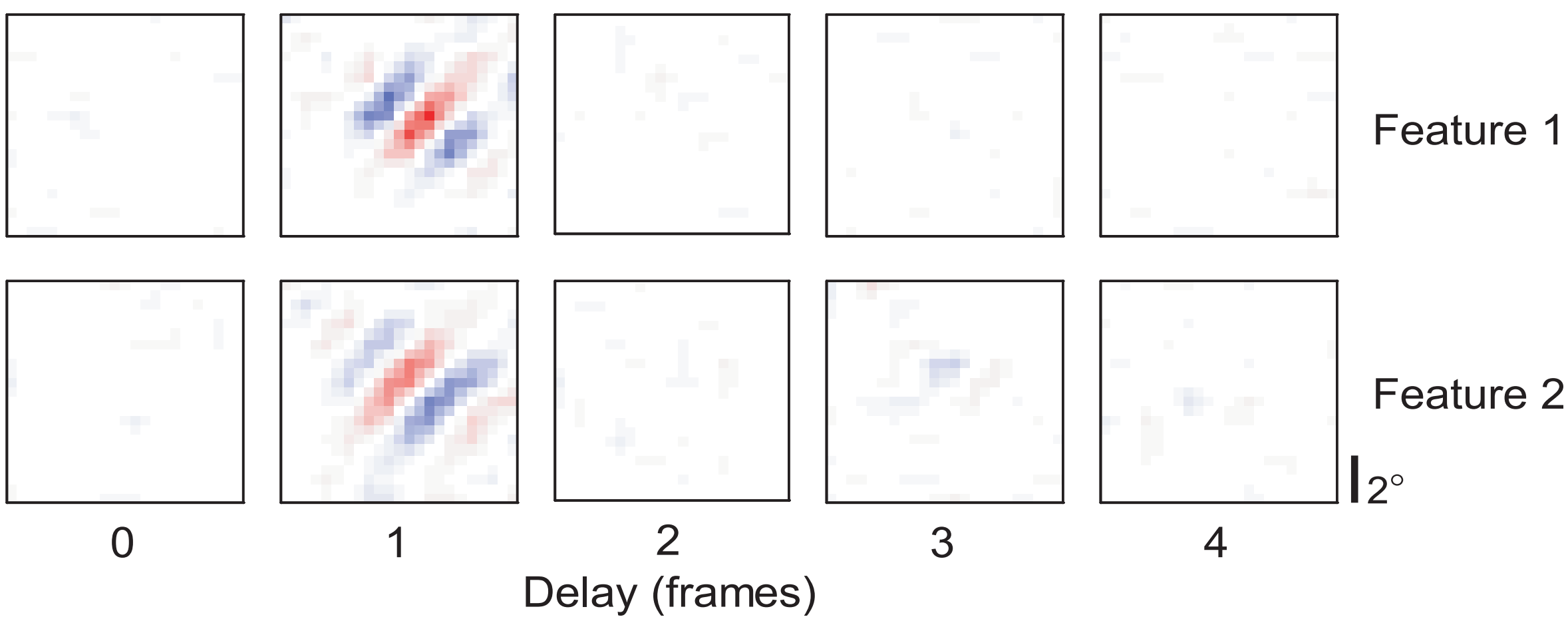

Supplementary Figure 2 Estimated spatiotemporal features of a complex cell. Note that the signals in the features are almost completely contained in the spatial feature at a delay of 1 frame.
